# Recurrent Transient Ischemic Attacks and Stroke Due to Carotid Dissection During Air Travel: A Case Report

**DOI:** 10.7759/cureus.34340

**Published:** 2023-01-29

**Authors:** Maricela Mendes, Ana Cristina Gonçalves, João Tarrio, Eduardo Rodrigues, Tatiana Luis, Dalila Perneta

**Affiliations:** 1 Family Medicine, Centro de Saúde de Machico, SESARAM, EPERAM, Funchal, PRT; 2 Family Medicine, Centro de Saúde do Estreito de Câmara de Lobos, SESARAM, EPERAM, Funchal, PRT; 3 Neuroradiology, Hospital Central do Funchal, SESARAM, EPERAM, Funchal, PRT; 4 Family Medicine, Centro de Saúde de Machico, SESARAM, EPERAM, Machico, PRT; 5 Family Medicine, Centro de Saúde de Machico, SESARAM, EPERAM, FUNCHAL, PRT

**Keywords:** aerospace medical association, carotid arterial dissection, air travel, stroke, transient ischemic attack

## Abstract

Carotid artery dissection causes 2.5% of ischemic acute strokes and is more common in younger rather than older patients. Extracranial lesions often manifest as transient and reversible neurological deficits until a stroke occurs. In this case, we describe a 60-year-old male patient with no known cardiovascular risk factors who experienced three transient ischemic attacks (TIAs) in four days while traveling in Portugal. At the emergency department, he was treated for an occipital headache associated with nausea and two episodes of decreased left upper-limb muscle strength lasting two to three minutes with spontaneous recovery. He requested discharge against medical advice so that he could travel home. During the return flight, he had a severe right parietal headache followed by decreased muscle strength in the left arm. After an emergency landing in Lisbon, he was referred to the local emergency department, where his neurological examination revealed preferential gaze to the right exceeding the midline, left homonymous hemianopsia, minor left central facial paresis, and spastic left brachial paresis. On the National Institutes of Health Stroke Scale, he scored 7. A head CT was performed, showing no acute vascular lesions (i.e., Alberta Stroke Program Early CT Score of 10). However, an image compatible with dissection was identified on CT angiography of the head and neck and confirmed by digital subtraction angiography. The patient underwent balloon angioplasty and placement of three stents in the right internal carotid artery with vascular permeabilization.

This case highlights how prolonged and incorrect cervical posture and microtrauma secondary to aircraft turbulence may be associated with carotid artery dissection in predisposed individuals. The Aerospace Medical Association guidelines advocate that patients with a recent acute neurological event should avoid air travel until clinical stability is assured. As TIA is considered a harbinger of stroke, patients should be properly evaluated and avoid air travel for at least two days after the event.

## Introduction

Carotid artery dissection, which represents 2.5% of the causes of ischemic acute stroke (IAS), is more common in young patients [1]. Extracranial lesions often manifest as transient and reversible neurological deficits until a stroke occurs [2]. About 31% of transient ischemic attacks (TIAs) lead to a stroke within 90 days [3]. Cervical arterial dissection occurs when the wall leaflets separate, causing intimal injury or rupture of the vasa vasorum with intramural hematoma formation. Dissection leads to the narrowing of the vessel lumen and the release of thrombogenic factors caused by intimal injury [1,2]. 

Carotid dissection can be caused by direct cervical trauma, sudden neck movements, high blood pressure, and smoking. Inherited connective tissue abnormalities such as Ehlers-Danlos type IV or Marfan syndrome also are associated with an increased risk of spontaneous dissection. Cystic necrosis of the media, pseudoxanthoma elasticum, alpha-1 antitrypsin deficiency, autosomal dominant polycystic kidney disease, hyperhomocysteinemia, and osteogenesis imperfecta are other intrinsic predisposing risk factors [3,4]. Aircraft cabins represent an environment conducive to dissection in predisposed individuals. This risk is exacerbated by incorrect cervical postures during flight and repeated cervical microtrauma secondary to turbulence [5,6]. This case highlights a rare and underdiagnosed cause of stroke and a possible contraindication for air travel among those with recurrent TIAs.

## Case presentation

A 60-year-old male born in the Autonomous Region of Madeira and currently living in Switzerland came to the Primary Care Health Center Emergency Service in Machico, Portugal on July 28, 2019, complaining of occipital headache with a constant, non-pulsatile intensity of 7/10 on the numeric rating scale, along with nausea and two episodes of decreased muscle strength in the left upper-limb lasting two to three minutes with spontaneous recovery. He had a personal history of degenerative bone disease of the lumbar spine and no known cardiovascular risk factors. He reported a similar previous episode of paresis three days before going to the emergency room. Upon observation, there were no changes in the neurological examination and the electrocardiogram was normal. After symptomatic therapy, he was referred to the emergency department for further evaluation.

At the Hospital Emergency Department in Funchal, the results of the objective examination were comparable to that performed at the Primary Care Health Center. Analytical and cranioencephalic CT imaging showed no acute vascular ischemic or hemorrhagic lesions (Alberta Stroke Program Early CT score (ASPECTS)=10). He was discharged against medical advice so that he could board a flight to Switzerland in the next few hours, with a prescription of acetylsalicylic acid (ASA), 150 mg per day.

**Figure 1:**
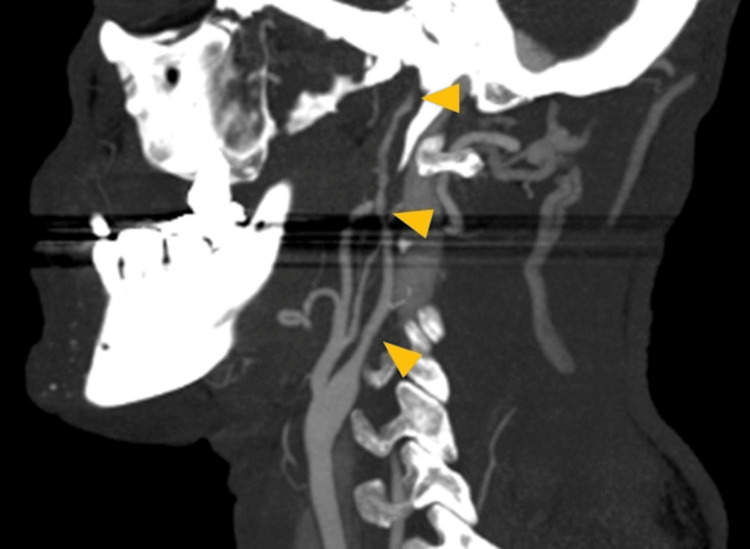
Sagittal CT angiography of the neck arteries showing irregular and filiform cervical segment of the right internal carotid artery consisted with dissection

**Figure 2:**
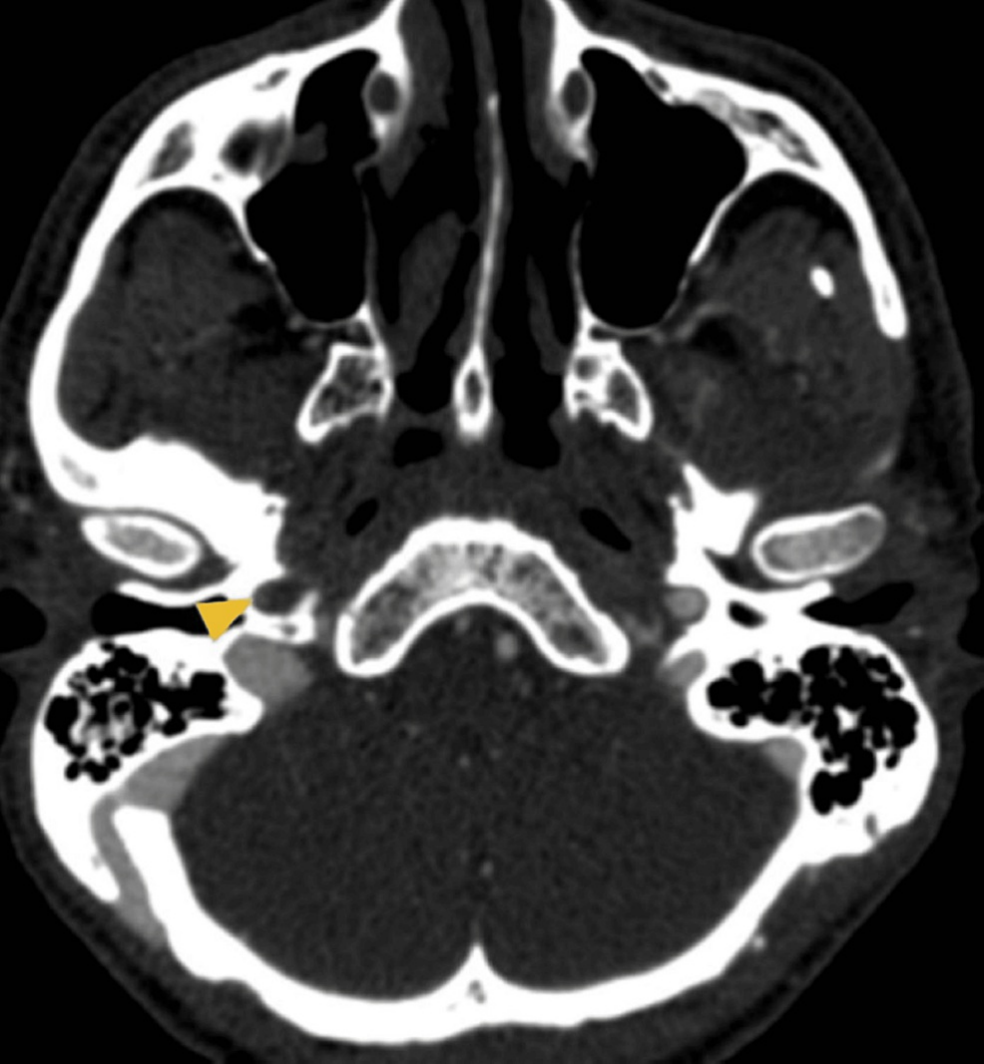
Axial cerebral CT angiography showing occlusion in cervical-petrous transition of the right internal carotid artery due to dissection

**Figure 3:**
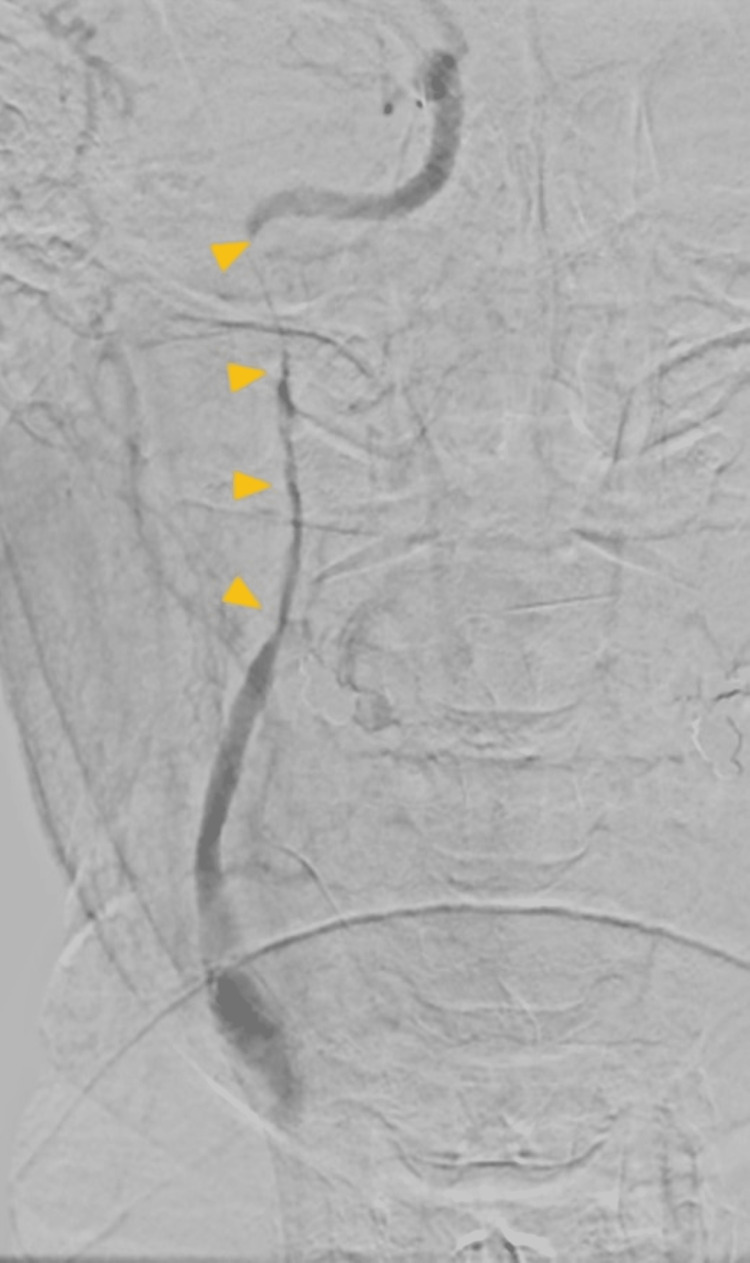
Digital subtraction angiography (frontal view) showing the dissection of the cervical segment of the right internal carotid artery, seen as a tapered lumen disappearing below the base of the skull

Seven hours after hospital discharge, on the return flight to Switzerland, he began to experience an intense right parietal headache followed by a decrease in upper-limb muscle strength. After an emergency landing in Lisbon, he was taken to the local emergency room, where the code stroke protocol was activated. He was alert, cooperative, and oriented to persons, time, and space. Neurological examination showed preferential gaze to the right exceeding the midline, left homonymous hemianopsia, minor left-central facial paresis, and spastic left brachial paresis (National Institutes of Health Stroke Scale=7). A CT angiography of the supra-aortic and cerebral trunks in the sagittal (Figure 1) and axial (Figure 2) planes was performed, showing an image compatible with arterial dissection, specifically an occlusion of the right internal carotid artery at the cervico-petrous transition by dissection of the cervical segment. The diagnosis was confirmed by digital subtraction angiography (Figure 3).

The patient underwent balloon angioplasty and placement of three stents in the right internal carotid artery with vascular permeabilization and was admitted to the cerebrovascular unit. During hospitalization, a transthoracic echocardiogram, electrocardiogram, and analytical evaluation showed no significant changes. Dual antiplatelet therapy (150 mg ASA once a day and 90 mg ticagrelor twice daily) and statin (40 mg atorvastatin once a day) were instituted. After 10 days of hospitalization, the neurological examination was unaltered (National Institutes of Health Stroke Scale=0), and the patient was discharged with the diagnosis of ischemic stroke in the territory of the middle cerebral artery in the context of occlusive dissection of the right cervical internal carotid artery. Dual antiplatelet therapy (150 mg ASA once a day and 90 mg ticagrelor twice daily) for six weeks was recommended, followed by 150 mg ASA indefinitely and a high-potency statin. Avoidance of air travel for one month and carotid Doppler ultrasound monitoring at zero, three, and six months after the event was recommended.

## Discussion

Stroke accounts for 2% of all medical emergencies during commercial flights, with an estimated incidence of 1:163,420 flights [7]. Prolonged and incorrect cervical posture and microtrauma secondary to aircraft turbulence may increase the risk of carotid artery dissection in predisposed individuals. The physical environment of the aircraft (hypoxia, reduced humidity, turbulence) itself also increases susceptibility to cerebral ischemia [5,7,8].

In this case, a 60-year-old male patient with no known cardiovascular risk factors experienced three episodes compatible with a TIA within four days while traveling in Portugal. He requested discharge against medical advice and while on a flight the following day, he developed an ischemic stroke with symptoms compatible with carotid artery dissection (e.g., central facial palsy, homonymous hemianopsia, unilateral headache, and preferential gaze). A history of successive TIAs with no known cardiovascular risk factors warrants an etiological study and measures to prevent a subsequent stroke.

Recommendations regarding air travel in patients with a recent ischemic event are scarce. As a general risk factor for stroke, air travel should be avoided [5,9,10]. The Aerospace Medical Association guidelines advocate that patients with a recent acute neurological event should avoid traveling until clinically stable, particularly due to the risk of psychomotor disability, the reduced ability to withstand the stressors inherent to a flight, and the risk of post-event complications [9]. As TIA is considered a harbinger of stroke, patients should be properly evaluated and avoid air travel for at least two days after the attack. In cases of stroke, patients with uncomplicated recoveries should wait five to 14 days and then receive supplementary oxygen when traveling in the first two weeks after the event [10,11]. A mid-flight stroke precludes the option of thrombolysis, effective intervention in blood reperfusion, and reversal of focal neurological deficits [5,9-11].

## Conclusions

The clinical case presented here highlights the importance of surveillance in patients experiencing recent TIA episodes, regardless of origin. These patients should postpone air travel until the etiology of the neurologic lesion can be identified and treated. Hypoxia, reduced humidity, turbulence, and incorrect cervical posture during air travel can aggravate pre-existing cerebral ischemia. Thus, to avoid adverse patient outcomes, air travel should be avoided after the event of TIA or stroke until clinical stability is assured.
